# “I would sooner die than give up”: Huxley and Darwin's deep disagreement

**DOI:** 10.1007/s40656-021-00409-3

**Published:** 2021-04-09

**Authors:** Mary P. Winsor

**Affiliations:** Institute for the History and Philosophy of Science and Technology, University of Toronto, 550 Spadina Crescent, Toronto, ON M5S 2J9 Canada

**Keywords:** History of systematics, History of taxonomy, Homology, Thomas Henry Huxley, Charles Darwin, William Whewell, John Stuart Mill, Richard Owen, Hugh Strickland, Lagostomus, Archetype

## Abstract

Thomas Henry Huxley and Charles Darwin discovered in 1857 that they had a fundamental disagreement about biological classification. Darwin believed that the natural system should express genealogy while Huxley insisted that classification must stand on its own basis, independent of evolution. Darwin used human races as a model for his view. This private and long-forgotten dispute exposes important divisions within Victorian biology. Huxley, trained in physiology and anatomy, was a professional biologist while Darwin was a gentleman naturalist. Huxley agreed with John Stuart Mill's rejection of William Whewell's sympathy for Linnaeus. The naturalists William Sharp Macleay, Hugh Strickland, and George Waterhouse worked to distinguish two kinds of relationship, affinity and analogy. Darwin believed that his theory could explain the difference. Richard Owen introduced the distinction between homology and analogy to anatomists, but the word homology did not enter Darwin's vocabulary until 1848, when he used the morphological concept of archetype in his work on Cirripedia. Huxley dropped the word archetype when Richard Owen linked it to Plato's ideal forms, replacing it with common plan. When Darwin wrote in the *Origin of Species* that the word plan gives no explanation, he may have had Huxley in mind. Darwin's preposterous story in the *Origin* about a bear giving birth to a kangaroo, which he dropped in the second edition, was in fact aimed at Huxley.

## Introduction

The connection between the classification of living things and evolution is generally assumed to be a rather simple business. Charles Darwin predicted in *The Origin of Species* (1859, p. 486) that after his theories are accepted, “Our classifications will come to be, as far as they can be so made, genealogical.” In response, the two leading champions of evolution, Ernst Haeckel and Thomas Henry Huxley, reimagined taxonomic groups as ancestors and descendants. Nowadays biologists view taxonomic groups of plants and animals as branches of the tree of life, our standard metaphor for past genetic relationships. Students these days are being taught that we can classify organisms thanks to information about their genome supplied by computers. There are many historical facts, however, that ought to challenge our assumption that the connection between evolution and classification is straightforward. Some of these facts are well known, such as that the system of classifying plants and animals still in use was founded by a pious eighteenth century botanist, Carl Linnaeus, who took the Book of Genesis literally. Another bit of history, overlooked until now, might be more revealing because religion was not involved. Two years before the publication of the *Origin,* Darwin privately discussed the nature of classification with Huxley. Darwin was certain that classification should reflect evolution, but Huxley insisted that a theory about how species can change could have no bearing on how they are classified. Finding that Huxley was adamant, Darwin confided to a mutual friend, “I would sooner die than give up” (Burkhardt & Smith, 1990, 7: 432). Darwin told Huxley that he had his views in mind when he was writing about classification in the *Origin*.

It is difficult to put ourselves into the shoes of biologists who worked before 1859, because our teachers, and their teachers, grew up in a world transformed by the effects of Darwin's book. Reading history, we cheer for the Huxley who shamed the Bishop of Oxford in 1860 for joking about our ape ancestry, we feel allegiance with the Huxley who proposed that birds evolved from dinosaurs, and we admire the Huxley who worked to expand the role of science in education. Yet that same Huxley, throughout his life, would have reacted with derisive laughter if anyone had said to him, “Nothing in biology makes sense except in the light of evolution.”^1^ He knew better, because his early biological research, carried out in the 1850s while he was scorning evolution as unscientific speculation, had not only made sense to him, it had also earned him a gold medal from the Royal Society of London. If we truly want to learn from the experience of our forebears, instead of cherrypicking the past for comforting reflections of our beliefs, we might start by noticing that Darwin's gold medal from that prestigious scientific body was awarded in 1853, a year after Huxley's. And let us please stop calling this influential person “Darwin's bulldog.” He may have said that about himself, but only once, in private, and no one else in the nineteenth century is known to have called him that (van Wyhe, 2019). This is a twentieth-century label that misrepresents not just Huxley's relationship to Darwin but his substantial and independent influence on the course of history.

Let us begin with a close inspection of the letters between Darwin and Huxley discussing classification. Paying close attention to their choice of words will give us clues to their thoughts.

## Their recorded disagreement, 1857–1859

After HMS *Beagle* returned to England in 1836, Charles Robert Darwin, a gentleman of independent means, immersed himself in the scientific life of London. By early 1837 he became convinced of evolution and the next year he worked out natural selection as its main cause. He moved to the rural village of Downe in 1842, where he continued to work on his theory.^2^ In 1844 the idea that life had evolved was given new prominence by a popular book, *Vestiges of the Natural History of Creation*; the anonymous author, Robert Chambers, published frequent revised editions. In 1850 Huxley, Darwin's junior by sixteen years, settled down permanently in London after sailing around the world as assistant surgeon on HMS *Rattlesnake.*^3^ Darwin rarely visited London, but he and Huxley knew of each other's publications and reputations. Many of Darwin's letters, and a few of Huxley's, have survived, so we can eavesdrop on their conversation.^4^

Evolution was still the talk of the town, because it seemed that science was threatening to undermine religion. Huxley, while no fan of orthodox Christianity, wrote a damning review of *Vestiges* in 1854 because he thought that its ill-founded speculation damaged the reputation of sober science. Huxley believed the fossil record did not support the idea that life had gradually developed from simple to advanced. In 1854 Darwin was just concluding eight years of work on barnacles (Cirripedia), identifying all known species and classifying them into genus and family. When he wrote to Huxley about this taxonomic project on September 2, 1854, he included a remark on evolution.

I have just been reading your Review of the Vestiges … but I cannot think but that you are rather hard on the poor author. I must think that such a book, if it does not other good, spreads the taste for natural science.―

But I am perhaps no fair judge for I am almost as unorthodox about species as the Vestiges itself, though I hope not quite so unphilosophical. (Burkhardt & Smith, 1990, 5: 213)

At this point Darwin had begun to sort his old notes about how species may have evolved; he surely worried that Huxley might write a hostile review of the book he planned to write.

On the subject of classification, Darwin's interests first intersected with Huxley's in 1855 over a question about the higher groups, meaning those above the rank of genus and species. In the system inherited from Linnaeus, every individual organism belongs to a species, every species belongs to a genus, every genus sits in an order, and each order must belong to a class.^5^ On the pages of Linnaeus's books, the running head is printed at the top, above the names of the species; the running head consists of the names of the class, order, and genus to which the species on the page belong, making those groups literally higher.^6^ Later taxonomists had an exploding number of species to catalogue, so they inserted more ranks, including family, sub-order, and sub-class. Huxley was preparing a course of lectures surveying invertebrate animals, so he was using Darwin's recent barnacle volumes. Darwin wrote a long letter to Huxley on March 8, 1855, clearly in response to an inquiry from Huxley that is now lost. Darwin had announced his judgement on where to rank barnacles in his title, *A monograph on the sub-class Cirripedia*. To call the group a sub-class of Crustacea agreed with the opinion of James Dwight Dana in the United States, while Henri Milne-Edwards in France had situated barnacles one rank lower down, under one of the main divisions of Crustacea, the Entomostraca.^7^ Darwin wrote to Huxley what he had already said in print, that he thought the similarity between Entomostraca larvae and barnacle larvae was merely “analogical” (1854, p. 18). He and Huxley both knew that taxonomists regarded analogies as isolated points of similarity that should be ignored in classification.

In his *Monograph* Darwin had also suggested that something strange was going on.

As far as concerns our present discussion on Cirripedes, the first three divisions [of Crustacea], as valued by Dana, will best serve as standards of comparison; but it is not unimportant to our present purpose, as showing how difficult it is to weigh the value of the higher divisions of a Class, to observe the wide difference in opinion of two naturalists, so eminent for their knowledge of the class in question and for their high abilities. (Darwin, 1854*,* p. 11)

By *our present purpose* he probably meant the task of making a classification. The issue of weighing the value of characters is of course of daily concern in the practice of taxonomy. Darwin repeated this sentiment in his 1855 letter to Huxley.

How difficult a subject is the classification of the higher groups in any class: see how Dana & Milne Edwards, such competent judges, differ! (Burkhardt & Smith, 1990, 5: 282)

We know of no response to this comment from Huxley.

In April of 1856, when Darwin was considering how to compose a convincing exposition of his theory of evolution by natural selection, he invited two taxonomists, the botanist Joseph Dalton Hooker and the entomologist Thomas Vernon Wollaston, to come stay with him for a weekend, and he also asked Huxley. All three of his guests already knew that Darwin believed that species were not fixed; Darwin wanted to see if he could nudge them towards his view. Besides spending hours indoors talking, Darwin took them into his garden to show them living examples of the remarkably different breeds of pigeon that hobbyists had created. A few weeks later Darwin outlined and began writing what promised to be a very long book.

Over a year later, in early autumn of 1857, Huxley sent Darwin a printed copy of his lectures (Huxley, 1857a). In response, on September 15, 1857, Darwin elevated his published *difficult* in the direction of *impossible*.

I have been glad to read what you say on the value of the Group [Cirripedia]; & I daresay you are right; but how difficult, not to say impossible it is to classify the higher groups. Take the Crustacea & see what differences in opinion in the half-dozen best judges, without much difference in the facts they go on. (Burkhardt & Smith 1990, 6: 454)

Hindsight allows us to guess with confidence why Darwin thought that the difficulty in classifying higher groups was significant. Early naturalists who catalogued forms of life had struggled with the question of continuity: when all the world's living things have been found, would nature be continuous, because transitional forms will fill in the gaps we now see? That would expose taxonomic categories as human inventions. Or will nature still be discontinuous, in which case natural groups will be confirmed as real entities? (Stevens, 1994). Darwin collapsed that longstanding dichotomy. In his vision of the history of life, the discontinuity seen today is real, and continuity is also real but is located in the past, where an unbroken stream of life stretches back countless millions of years, leaving us only a few remnants as fossils. What we call a taxonomic group is a collection of species that are literally kin. Species in a genus have a close blood relationship while species in a higher group are also related by blood, but the line of connection reaches even further into the past. This was a version of evolution which few of his contemporaries had yet imagined. It means that classification must be both natural and man-made at the same time.^8^ It is impossible to chop groups out of a continous stream without making arbitrary choices. Darwin envisioned the forces of change as irregular, so that species today have irregular degrees of relatedness. The fact that taxonomists struggled to define species was to Darwin not a problem but a clue. Many species are easy to distinguish, while other species elude definition, and this to him was evidence that for some species, a process of change is going on now, while for other species, change happened in the past. Groups above the level of species were grist for his mill in the same way. The fact that eminent taxonomists could disagree about the higher groups, Darwin took as evidence for his theory.

Huxley's reply to Darwin's September 15 letter has been lost, but it apparently contained a direct question about Darwin's views on classification. Darwin told Huxley on September 26, 1857, that he believed the natural system expresses ancestry.

In regard to Classification, & all the endless disputes about the Natural System which no two authors define in same way, I believe it ought, according to my heteredox notions, to be simply genealogical.— But as we have no written pedigrees, you will, perhaps, say, that this will not help much; but I think it ultimately will, whenever heteredoxy becomes orthodoxy, for it will clear away an immense amount of rubbish about the value of characters &— will make the difference between analogy & homology, clear— The time will come I believe, though I shall not live to see it, when we shall have very fairly true genealogical trees of each great kingdom of nature.^9^ (Burkhardt & Smith, 1990, 6: 456)

Undoubtedly the word *pedigree* had been part of the conversation during that weekend in Downe the previous year, because keeping a record of bloodlines was a tool as important for pigeon fanciers as for racehorse breeders. The word *genealogical* is particularly significant, and we will return to it.

Huxley sent a prompt response, but the beginning of his letter has been lost. Darwin saved part of it in his file on classification.

Cuviers definition of the object of Classification seems to me to embody all that is really wanted in Science— it is *to throw the facts of structure into the fewest possible general propositions*— This of course leaves out of view & passes by, all questions of pedigree & possible modifications— dealing with existing animals and plants as faits accompli

I for one believe that a *Scientific & logical Zoology* & Botany are not at present possible— for they must be based on sound Morphology— a *Science* which has as yet to be created out of the old Comparative Anatomy — & the new study of **Development** [embryology] When the mode of thought & speculation of Oken & Geoffroy S. Hilaire & their servile follower Owen, have been replaced by the principle so long ago inculcated by Caspar Wolff & Von Baer & Rathke— & so completely ignored in this country & in France up to the last ten years— we shall have in the course of a generation a science of Morphology & then a Scientific Zoology & Botany will flow from it as Corollaries—

Your pedigree business is a part of Physiology— a most important and valuable part— and in itself a matter of profound interest— but to my mind it has no more to do with pure Zoology— than human pedigree has with the Census— Zoological classification is a Census of the animal world (Burkhardt & Smith, 1990, 6: 461–462)

The British census was a massive project of collecting data every decade with which Huxley and Darwin were familiar, not just because of the 1851 count but because William Farr, the commissioner who was turning the census into a tool for public health, had recently joined them as a member of the Royal Society. Huxley was using *census* as a metaphor, where we might use *snapshot*, to suggest a theory-free record of facts. Though it would be easy show that pinning down facts about millions of people cannot be perfectly objective, we need not perform this philosophical exercise, since Huxley did not use the comparison again.^10^ The word *census* can serve us as a label for these three paragraphs of Huxley's.

The reference to Georges Cuvier was surely to *Le Règne animale*, which first appeared in 1817 and was thereafter continually expanded, updated, and translated. Huxley's friend William Benjamin Carpenter worked on an edition that was published in London in 1849. The first page starts with Cuvier's declaration that “for nearly thirty years … the constant aim of my labour has been to reduce it [the science of comparative anatomy] to general rules, and to propositions that should contain their most simple expression.”^11^ In the *Origin of Species* (p. 413) Darwin would mention that “some authors” consider the natural system as “an artificial means for enunciating, as briefly as possible, general propositions.” He may have had in mind Huxley's Cuvier, although there were many other writers who had expressed the same idea.

We must set aside for the moment the five other names listed in the census letter, because Huxley's view of what is wanted in science, and what would make botany and zoology scientific, differs so widely from today's that his middle paragraph will require careful unpacking. Darwin's reply ignored that heavy paragraph and brushed aside the mention of Cuvier, eminent but dead, in favour of “most naturalists.” Darwin wrote on October 3, 1857,

I know you have no time for speculative correspondence; & I did not **in the least** expect an answer to my last. But I am very glad to have had it, for in my eclectic work, the opinions of the few good men are of great value to me.—

I knew, of course, of the Cuvierian view of Classification, but I think that most naturalists look for something further, and search for “the natural system”,— “for the plan on which the Creator has worked &c &c.—It is this further element which I believe to be simply genealogical.

But I should be very glad to have your answer (either *when we meet* or by note) to the following case, *taken by itself & not allowing yourself to look any further than to the point in question*.

*Grant* all races of man descended from one race; grant that all structure of each race of man were perfectly known— **grant** that a perfect table of descent of each race was perfectly known—grant all this, & then do you not think that most would prefer as the best classification, a genealogical one, even if it did occasionally put one race not quite so near to another, as it would have stood, if allocated by structure alone. Generally, we may safely presume, that the resemblance of races & their pedigrees would go together.

I should like to hear what you would say on this purely theoretical case.^12^ (Burkhardt & Smith, 1990, 6: 462–463)

Darwin suggested Huxley give his reply “when we meet,” presumably referring to November 19, 1857, when they would see each other in London at the Philosophical Club of the Royal Society (Burkhardt & Smith, 1990, 6: 485n8).

It may seem odd that when the topic was the classification of the entire diversity of life, these men both chose to cite our own species, Darwin using “all races of man” and Huxley the census. Darwin avoided the topic of human evolution in the *Origin*, not addressing it until his *Descent of Man* in 1871. It is easy to imagine that the history of thought unfolded in logical progression, moving from considering the evolution of plants and animals on to the evolution of *Homo sapiens*. In fact the inference has always worked in the other direction. Differences among humans, both within families and between races, offer us a model for understanding domestic breeds and other living things. Darwin knew that Huxley had neither interest in, nor experience with, how farmers produce different varieties of plants and animals. He also knew that he and Huxley shared the experience of seeing strikingly different kinds of people during their trips around the world. Whether humans constitute one biological species or several was entangled with the moral and political issue of slavery, which had been outlawed throughout the British Empire in 1834 but was still being hotly debated at this time in the United States. Liberal thinkers believed that all races of humans originated from one common ancestor, but this was denied by some defenders of slavery.

The “purely imaginary case” of human pedigree that Darwin put to Huxley on October 3, 1857, found its way into the *Origin*, where it ended a paragraph about varieties of pigeons.

If it could be proved that the Hottentot had descended from the Negro, I think he would be classed under the Negro group, however much he might differ in colour and other important characters from negroes. (Darwin, 1859, p. 424)

Both of these terms for indigenous people in South Africa have become offensive, but Darwin was using names he learned not only from books in the *Beagle*'s library and but also from local people he met when the ship visited the Cape of Good Hope. There he had hired a Khoekhoen man, who spoke excellent English, to be his guide for a three-day ride across the countryside near Cape Town (Darwin, 1839, p. 576). Thus Darwin knew first-hand about people who differed from one another by language, culture, and physical features. Although Darwin made the strategic decision not to deal with our own species in the *Origin*, this sentence exposes the fact that human races had always been for him good examples of variation within a species. He deleted the sentence in the 1869 edition of the *Origin*.

In choosing the words *genealogy* and *pedigree,* Darwin was trying to ease Huxley into his mode of thought. Both words have two distinct but connected meanings, one physical and one immaterial. They can mean a written record, a list or chart of names on paper, a chronicle of births, marriages, and children, that make up a sequence of ancestors and descendants, or these words can mean those relationships, which are as immaterial as all history is, not tangible but still real. Every person has a great-great grandmother, even if some people have no record of her name. The word *pedigree* was already being applied to the bloodline of domestic animals, but *genealogy* normally referred to human ancestry. When Darwin told Huxley that classification should be *genealogical*, he was compressing his entire evolutionary theory into a single metaphor.

Darwin's background was radically different from Huxley's. Darwin's thinking had been shaped by the scientific reasoning of Charles Lyell's *Principles of Geology*. The subtitle of that book states Lyell's method: *Being an attempt to explain the former changes of the earth's surface, by reference to causes now in operation*. Geology presents the subtle challenge of understanding the distant past, a project essentially different from sciences like chemistry that ignore history. Lyell said we should not just record minerals and measure valleys, we must study how rivers are eroding hills and how an exploding volcano deposits ash and lava. Darwin had successfully applied Lyell's method to Pacific atolls, which he explained by the action of countless tiny coral polyps, combined with relative changes in sea level. Darwin confronted the question of evolution by the same method: he looked for causes not in the fossil record but in the way plants and animals are varying now. When Darwin wrote to Huxley “we have no written pedigrees,” he was asking him to realize that even without a physical record, we can be sure that every living thing has a real ancestry. For twenty years Darwin had been living with the belief that a species can not only change but divide, so that its descendants could belong to two or more different species and, given enough time, those could give rise to numerous species that we would call a higher group. Nothing in Huxley's education or experience prepared him for this. For Huxley, to use the fact that a species can split into varieties as an explanation for the existence of higher groups was mere speculation, while for Darwin it was an extension of Lyell's method.

The next letter we have from Darwin to Huxley, a month after their meeting in London, contains Darwin's comments on a document Huxley was about to publish.^13^ Huxley's task was to explain to students the meaning of the fossils catalogued in the Museum of Practical Geology (attached to the School of Mines in London). Darwin's letter is dated December 16, 1857.

In my opinion your Catalogue is simply the very best Resume by far, on the whole Science of Natural History, which I have ever seen.

I really have no criticisms; I agree with every word. Your metaphors & explanations strike me as *admirable*. In many parts it is curious how what you have written agrees with what I have been writing, only with the melancholy difference for me that you put everything in twice as striking a manner, as I do.^14^

I append more for the sake of showing that I have attended to the whole, than for any other object, a few **most** trivial criticisms.

I was amused to meet with some of the arguments, which you advanced in talk with me, on classification; & it pleases me, as my long proses were so far not thrown away, as they led you to bring out here some good sentences.

But on classification I am not quite sure that I yet wholly go with you, though I agree with every word you have here said. (Burkhardt & Smith, 1990, 6: 505)

Huxley's text was already set in type when Darwin read it, so we may assume the published version is close to what Darwin read in 1857.^15^ The words with which Darwin agreed doubtless included Huxley's clear descriptions of the difference between artificial and natural systems, the difficulties naturalists faced in defining species, and what sorts of evidence counted for or against the mutability of species. Huxley was already vigorously stating the position for which he would become famous after the *Origin*: that the question of transmutation must be decided purely based on scientific evidence, free from any religious influence.

Darwin must have been especially gratified to read Huxley's statement:

The phrase “family resemblance” … perhaps, expresses better than any other, the sort of likeness which exists amongst the members of a natural group; specific and generic alliance having the same sort of relation as brotherhood and cousinhood. (1857b, p. 134)

At the very heart of Darwin's work was the conviction that the relationships that taxonomists express in their categories do not differ in kind, merely in degree, from the blood relationships we recognize within human families.

In spite of Darwin's declaration that all the comments he was sending were quite trivial, he did register an interesting objection. Huxley, to emphasize that classification should consider only anatomy (inert structure) and not physiology (living activity), offered a thought experiment.

If all forms of living beings were fossil, and we knew nothing about life, the natural classification of animals and plants would be exactly what it is now; except as it might be affected by the resulting deficiencies in our knowledge. (1857b, p. 135)

To this Darwin commented:

I do not understand how you can say that if only fossils existed there would be no difficulty in practically [classifying] species— as there is variation among fossils, as with recent, there seems to be same difficulty in grouping. (Burkhardt & Smith, 1990, 6: 506)

Darwin was speaking from hard-earned experience, for his monograph on barnacles included study of all the barnacle fossils then known, and Darwin had highlighted their great variability. This is a headache for a taxonomist, Darwin said, but it adds “one more to the many known proofs of the exhaustless fertility of Nature in the production of diversified yet constant forms” (1851a, p. 2). Darwin's comment shows us, however, how thoroughly he and Huxley were misunderstanding each other. Huxley's odd supposition (what if we knew nothing about life?) reflected two categories of study: anatomy deals with structure and physiology deals with function. This helps us understand what Huxley meant in his census letter. Darwin's *pedigree business* belonged to physiology because reproduction is a vital function of living things. For Huxley, classification should only consider the features of dead specimens.

Having declared that he agreed with every word, why did Darwin say, “But on classification I am not quite sure that I yet wholly go with you …”? Surely because of these two sentences in Huxley's paper:

But it is important to remember that the classification of animals and plants stands on its own basis and is entirely independent of physiological considerations. For the purposes of the classifier it is wholly immaterial whether, as some maintain, “species” are immutable, and have taken their origin independently of one another, directly from the hand of the Creator; or whether, as others think, they are indefinitely modifiable, and have all resulted from the changes induced by external influences upon some common stock. (1857b, pp. 134–135)

The essence of Huxley's disagreement with Darwin about classification was this insistence that classification must “stand on its own basis … entirely independent” of evolution.

In August 1858, which was eight months after this exchange, Darwin stopped working on the long exposition of his theory that he had been writing since 1856. Alfred Russel Wallace's discovery of the principle of natural selection impelled Darwin to condense his draft down to one volume, provisionally entitled “An Abstract of an Essay on the Origin of Species.” He was composing the penultimate chapter*,* Chapter XIII, which included his main discussion of classification, when he wrote to Huxley on March 13, 1859.^16^

I entirely agree with your remarks on Agassiz's Essay on Classification: it is all utterly impracticable rubbish, about his grades &c. &c. But, alas, when you read, what I have written on this subject, you will be just as savage with me. (Burkhardt & Smith, 1990, 7: 262)

Louis Agassiz had argued that the naturalness of the Linnaean system was evidence that the Creator had worked out His ideas in layers. Agassiz proposed a thought experiment, claiming that if God had created an animal of a unique sort, the sole member of its kind, taxonomists would still have to classify it into its own genus, order, and class, because each taxonomic rank expresses a different aspect of its nature (Winsor, 1991, p. 21).

On October 15, 1859, while preparing to send copies of the newly-printed *Origin* to colleagues, Darwin wrote to Huxley:

I shall be **intensely** curious to hear what effect the Book produces on you. I know that there will be much in it, which you will object to …. …The penultimate chapter, though I believe it includes the truth, will I much fear make you savage. (Burkhardt & Smith, 1990, 7: 350)

A week later Darwin told his closest friend, Hooker, about his disagreement with Huxley.

I am intensely curious to hear Huxley's opinion of my Book: I fear my long discussion on classification will disgust him; for it is much opposed to what he once said to me. (Burkhardt & Smith, 1990, 7: 356)

On November 23, 1859, Huxley wrote Darwin praising the book and pledging his support, while withholding full assent to Chapter XIII. He said that this chapter.

contains much that is most admirable, but on one or two points I enter a caveat until, I can see further into all sides of this question. (Burkhardt & Smith, 1990, 7: 390-391)

To this Darwin replied two days later:

On classification I fear we shall split. Did you perceive argumentum ad hominem Huxley. about Kangaroo & Bear (Burkhardt & Smith, 1990, 7: 399)

In the *Origin* Darwin asks his readers to imagine that an ancestral species of bear has given rise to several descendant species, and that in addition to giving rise to several new kinds of bears, one line of its descendants has gradually evolved into a kangaroo. This strange conjecture concludes a paragraph that began by arguing that naturalists already prefer genealogy over appearance when they classify males and females together into one species. He wanted his readers to extend that principle above the species level up to higher groups: genera and families.

But it may be asked, what ought we to do, if it could be proved that one species of kangaroo had been produced, by a long course of modification, from a bear? Ought we to rank this one species with bears, and what should we do with the other species [of kangaroo]? The supposition is of course preposterous; and I might answer by the *argumentum ad hominem*, and ask what should be done if a perfect kangaroo were seen to come out of the womb of a bear? According to all analogy, it would be ranked with bears; but then assuredly all the other species of the kangaroo family would have to be classed under the bear genus. The whole case is preposterous; for where there has been close descent in common, there will certainly be close resemblance or affinity. (Darwin, 1859, p. 425)

The vivid coming-out-of-the-womb scenario compresses into a single generation his theory that new species arise “by a long course of modification.” The consequence would be that taxonomists would have to reclassify this new kangaroo as a species in the bear genus, but that would force them to reclassify other kangaroos and wallabies with bears too.

The Latin term *argumentum ad hominem* nowadays is usually taken to mean an argument against a particular individual, an attempt to discredit one's opponent by impugning his character, but in the nineteenth century its original classical meaning was known (Hitchcock, 2017). Darwin and his readers understood it to mean an argument not based on law or logic but appealing to common sense, such as when a debater asks their opponent how they would judge another case similar to the one in question. This was respected as an effective rhetorical move rather than disparaged as a logical fallacy; it was certainly not rude. The term fits the hypothetical case Darwin had put in his letter to Huxley in 1857, since we humans care not just about our appearance but also about our ancestry. The instant Darwin uttered the bear-kangaroo story he immediately called it “preposterous; for where there has been close descent in common, there will certainly be close resemblance or affinity” (p. 425).^17^ He dropped the story from all subsequent editions of the *Origin*.

On December 14, 1859, Darwin again confided to Hooker:

I shall be very curious to hear what you think on my discussion on Classification in Ch. XIII; I believe Huxley demurs to the whole; & says he has nailed his colours to the mast, & I would sooner die than give up, so that we are in as fine a frame of mind to discuss the point, as any two religionists. (Burkhardt & Smith, 1990, 7: 432)

These *religionists* were clerics in the 1840s and '50s who exchanged passionate essays debating theology and ritual within the Church of England. The verb *demur* means refusal to assent, which is not outright denial. Because a battleship's *colours* is a flag attached to a halyard, so it can be raised or lowered, the metaphor of fixing it to the mast means absolute refusal to surrender.

## Distinct realms: taxonomy and morphology

Huxley's 1857 disagreement with Darwin exposes a powerful division at work in the biological sciences at the time. Morphology, which Huxley called “a science yet to be created,” was an approach that belonged to anatomy, solidly based in the ancient profession of medicine, while taxonomy was a practice central to natural history, which had more diffuse institutional connections. The word *classification* that sat at the centre of their conversation names a human activity that can have almost any object (minerals, clouds, books, words). With respect to the classification of plants and animals, Darwin's thoughts turned to “most naturalists” and “authors who dispute about the natural system,” that is, to people who practiced taxonomy, also known as systematists. Huxley was coming to the conversation from a different background. He had been trained in physiology and anatomy and had pledged his heart to morphology. To understand why these two intelligent scientists found themselves in deep disagreement, we must recognize the intellectual and social landscapes containing those distinct realms.^18^

The term *natural history* covered several fields still using that name, including knowledge of the life history of plants and animals, as well as topics that were well on the way to becoming recognized disciplines, such as geology and palaeontology. Probably the majority of people calling themselves naturalists were engaged in taxonomy, which involved collecting and preserving specimens of plants and animals and describing, naming, and publishing their findings (Endersby, 2018). There was enough work to be done that naturalists were already specializing, as signalled by words like *botanist*, *zoologist*, *ornithologist*, or *entomologist*. The word *biology*, invented at the start of the century, was not yet widely used, and professorships in *natural history*, such as the one that Huxley applied for in 1851 at the University of Toronto, could cover a variety of topics, including anatomy and physiology. Such positions were few, however, and most naturalists pursued it as a hobby, as Darwin had done when he was a student. Years later he recalled, “No pursuit at Cambridge was followed with nearly so much eagerness or gave me so much pleasure as collecting beetles. It was the mere passion for collecting, for I did not dissect them and rarely compared their external characters with published descriptions” (Barlow, 1958, p. 62). Some people cared mostly for the beauty or rarity of their specimens while others became serious investigators and were called *philosophical* naturalists. The growing numbers of such men and women created a market for publications ranging from expensive folios with colour illustrations to cheap catalogues. Botany had long had a foothold within medical teaching because drugs came from plants, but Linnaeus in the eighteenth century had worked to convince governments that broader knowledge of the natural world was valuable to a nation's economy. By the nineteenth century there existed several state-supported museums and botanical gardens, creating employment for taxonomists. Nevertheless, most naturalists throughout the reign of Queen Victoria were amateurs.

The academic subjects of anatomy and physiology had been essential to the medical profession for centuries, which gave them institutional and intellectual advantages over natural history. At the dawn of the nineteenth century Cuvier had argued that comparative anatomy offered the equivalent of experiment to reveal physiological functions, so that close study of the entire animal kingdom, including obscure marine creatures, might ultimately benefit medicine. Rhetoric like this helped the natural history museum in Paris survive radical changes in the nation's government; by the same logic England's Royal College of Surgeons was willing to support a museum of comparative anatomy (the Hunterian) that employed Richard Owen, whose rise to fame began with the dissection of a deep-sea invertebrate, the pearly nautilus. This link between natural history and medicine explains why the captain of the *Rattlesnake*, a naval vessel, would encourage Huxley, a young assistant surgeon, to study the microscopic anatomy of jellyfish.

Darwin, sailing as an amateur naturalist, collected numerous specimens of animals, plants, and fossils, well aware that there were taxonomists back home who would describe whichever ones turned out to be new to science. Upon his return in 1836, Darwin's suspicions that species can evolve were confirmed when an ornithologist, John Gould, pronounced as good species some birds that Darwin had assumed were mere varieties. Within a few years Darwin was so well respected in the community of London naturalists that ornithologist Hugh Strickland invited Darwin to serve on a committee to standardize how zoologists should create names. An important lesson from that experience was that taxonomists who could not agree about whether species and genera actually exist could nevertheless agree on a useful reference system to denote them (McOuat, 1996).

Huxley, in contrast, began his career with no interest whatsoever in finding new species nor in collecting specimens. As a medical student, self-educated in French and German, he was excited by two novel approaches to comparative zoology: morphology, the abstract analysis of structure, and embryology, the study of individual development. Because Huxley, like Darwin, sailed around the world, it is easy for us to imagine their experiences were similar, but his later testimony “I am afraid there is very little of the genuine naturalist in me” (Huxley, 1900, p. 7) is fully confirmed by every detail we have of his early career. At sea he used a net to capture floating animals to study with his microscope, but his observations of the natural world were minimal and he formed no collections. His study of the cell layers of jellyfish was an exercise in pure morphology. He began his prize-winning article by disparaging zoologists who “contented themselves with stating matters of detail concerning particular genera and species, instead of giving broad and general views of the whole class, considered as organized upon a given type” (Huxley, 1849, p. 413). This disparagement of taxonomists who classify species in favour of anatomists offering “broad and general views” can be traced back to Cuvier's “old comparative anatomy” (as Huxley would call it in his census letter). Cuvier was famous for the attention he gave to higher groups, most famously the division of the animal kingdom into four principle *embranchements* or *plans*. When Huxley said that a class is “organized upon a given type,” his choice of words signalled his commitment to morphology, for its central idea was that living forms are not just compared to one another but are referred to a generalized abstraction, which was called the *type* or *archetype*. Owen, the leading British champion of morphology, complained that its central concepts, homology and archetype, were not appreciated by “the actual cultivators of Natural History in this country” (1849, p. 4). All those anatomists studied the bodies of adult animals, but Caspar Friedrich Wolff, Heinrich Rathke, and Karl Ernst von Baer, whom Huxley listed in that census letter, had enlarged anatomy by tracing how a fertilized egg develops (“evolves”), taking shape as it grows. During the *Rattlesnake* voyage, Huxley had considered von Baer's ideas in relation to classification of the higher groups in the embranchement Radiata (Winsor, 1976). In 1853 Huxley translated and published “Fragments relating to philosophical zoology, selected from the work of K. E. von Baer” and wrote an introduction complaining that British biologists' ignorance of German made them overlook the important discovery that there are four patterns of embryological development which coincide with, and explain, Cuvier's four principle forms (Huxley, 1853b, p. 176).

In 1853 Huxley applied the morphological method, which he had used so successfully on the Radiata, to another embranchement, Mollusca. When Darwin thanked Huxley for sending him a copy of this article, he confessed, “I have read it all with much interest, but it would be ridiculous in me to make any remarks on a subject on which I am so utterly ignorant …” (Burkhardt & Smith, 1990, 5: 133). When Huxley praised Darwin for his “beautiful and complete” monograph on barnacles, he alluded to the fact that Darwin was a naturalist, saying the quality of the barnacle work was “the more remarkable as proceeding from a philosopher highly distinguished in quite different branches of science, and not an anatomist *ex professo* (1857a, p. 238n)*.*”^19^

Historians have described how greatly the position of science changed in the nineteenth century as the needs of industry and government opened up jobs that required technical expertise. The process involved increasing financial support, social status, and other factors, but giving it a label like professionalization can imply that these developments happened by themselves, like growth. They did not. People who believed that science was essential to a more liberal and just future devoted passionate effort into forcing change. Britain's venerable Society for the Encouragement of Arts, Manufacturers and Commerce was working to expand the teaching of science in elementary education, and it commissioned speakers to promote this goal (Layton, 1973). In 1854 it organized a series of lectures, including “On the relation of chemistry and physics to other branches of knowledge” and “On the relation of the science of botany to other branches of knowledge.” Huxley was hired by the Society of Arts to speak “On the relations of physiological sciences to other branches of knowledge” (Anon., 1854).

Sticking to his assignment, Huxley used examples from physiology, such as the circulation of blood, saying little about either morphology or natural history. With the same rhetorical energy that later made him such a powerful supporter of evolution, Huxley set out to elevate the status of biology. How wrong it is to call biology an “inexact science,” he maintained, as if it were inferior to the “exact sciences” (physics, chemistry, and mathematics). By no means! Its methods “are obviously identical with those of all other sciences.” And so are its results.

If I say that respiration is performed by the lungs; that digestion is effected in the stomach; that the eye is the organ of sight; that the jaws of a vertebrated animal never open sideways, but always up and down; while those of an annulose [arthropod] animal always open sideways, and never up and down — I am enumerating propositions which are as exact as anything in Euclid.^20^ (1854, p. 14)

This appeal to the father of geometry implied that biology could someday be a deductive science, which may seem, to us in the twenty-first century, a strange expectation. We are heirs to a number of intellectual revolutions, including non-Euclidean geometry, quantum physics, and evolution by random mutation and natural selection, all of which were beyond Huxley's wildest dreams in 1854. We must keep this in mind if we hope to understand Huxley's side of his disagreement with Darwin. Although the living world Huxley envisioned was not the miraculously-created world of pious naturalists, neither was it like ours, nor like Darwin's, for they both include essential unpredictability. Huxley told his audience, “The Biologist deals with a vast number of properties of objects, and his inductions will not be completed, I fear, for ages to come; but when they are, his science will be as deductive and as exact as the Mathematics [sic] themselves.” (1854, p. 23).

In July of 1854, Huxley, much to his relief, found full-time employment, as lecturer on natural history at the Government School of Mines. Three years later his census letter would tell Darwin of his hope that botany and zoology would become “scientific and logical” at some future time. Huxley regarded what most naturalists were doing, classifying species, as not merely different from physiology and anatomy but scarcely deserving to be called scientific at all. The biology textbooks he would later write consisted mostly of anatomy and physiology.

## Affinity, homology, and analogy: ownership of words and ideas

When Darwin wrote in his 1857 letter to Huxley that his theory “will make the difference between analogy & homology, clear,” he was mixing concepts that came from two different realms: analogy from natural history and homology from morphology. Those topics remained distinct in the *Origin*, where analogy is discussed under the heading of Classification (pp. 427–428), homology under the heading of Morphology (pp. 434–439). Darwin's original and powerful concept of branching evolution, which he conceived in 1837, was founded almost exclusively in natural history (Winsor, 2009). In 1842, when his other original idea of natural selection was well enough developed, Darwin drafted a private sketch of his theories. It included his early insight that the conviction many taxonomists had arrived at, that there exists a natural system, could be explained by the opposing forces of inheritance, variation, and extinction. The relationship between members of a group in the natural system was termed by naturalists *affinity*, while isolated similarities between members of groups distant in the system were called *analogies*. In this 1842 sketch, Darwin's example of analogy was a South American mammal [*Chironectes minimus*] somewhat like the European otter in its aquatic lifestyle, long body, and webbed feet; its reproductive system, however, puts it far from true otters, which are placental, and squarely into the marsupial order. Cases like that show us the “true relationship of organisms,” Darwin believed. “Naturalists cannot avoid these terms of relation and affinity though they use them metaphorically. If [these terms are] used in simple earnestness the natural system ought to be a genealogical [one].” (Darwin, 1909, p. 36) The word *homology* is conspicuously absent from this 1842 sketch. The general idea is present, though, under the name of *unity of type*.

A year later, in 1843, Darwin's friend George Waterhouse asked for his opinion about some taxonomic decisions. Darwin replied by explaining his belief that “all relations of analogy &c &c &, consist of those resemblances between two forms, which they do not owe to having inherited it, from a common stock.” (Burkhardt & Smith, 1990, 2: 376) Forms related by affinity were those descended from a common ancestor. Here again, the word *homology* is absent, as it is from the enlarged sketch of his theory Darwin wrote in 1844. The earliest record of Darwin using that key morphological word (in plural form) is a letter he wrote Hooker in October 6, 1848, when Darwin was comparing the anatomy of barnacles to other crustaceans.

I have been delighted of late in having made out minutely the metamorphoses & consequently without any theory the homologies with ordinary crustacea. The shell of a Balanus, & even the whole peduncle & shell of Lepas is **certainly** the 3 anterior segments of the head, wonderfully modified & enlarged so as to receive the 14 succeeding cephalic, thoracic & abdominal segments; I declare I know of no more surprising metamorphosis, & it is *perfectly* clear & evident.— (Burkhardt & Smith, 1990, 4: 169)

Because embryonic barnacles have clearly defined segments, one can use the stages of moulting larvae to identify, “without any theory,” the parts of the adult rock barnacle (Balanus) and gooseneck barnacle (Lepas) that correspond to segments of other crustaceans. In the published monographs, Darwin compared these barnacles to the “archetype Crustacean” (1854, p. 16). Thanks to his labour with these marine invertebrates, Darwin could address Huxley in 1857 as a card-carrying morphologist.

Back in 1843 Darwin had been discussing classification with Waterhouse as a fellow naturalist, and in that community, the word *homology* (used in geometry to compare shapes) did not yet exist. What did exist was a special, recently-invented, meaning of the ordinary word *analogy*, previously used for any sort of comparison. The pious entomologist William Sharp Macleay announced in 1819 that relations of affinity were distinct from relations of analogy. The common English word *affinity*, long used to describe degrees of family kinship as well as for which chemicals will combine, was already being used to mean the relationship between members of a group in a natural classification. In Macleay's scheme, every taxonomic group has exactly five members, related by affinity and arranged in a circle. Analogy, he insisted, was a different sort of relationship in the divinely created order; analogy links members of different groups. Darwin and Waterhouse were among the many naturalists who had found Macleay's ideas worth considering in the 1820s and '30s.

After twenty years of debate had eroded support for Macleay's rigid system of numerical and geometric regularity, Strickland attacked it with two critiques in 1840 and 1841. He attacked the system but retained the crucial distinction between affinity and analogy. The natural system must use only affinities, he insisted, to express the Creator's underlying plan, whereas other similarities may either be accidental or demanded by circumstance (Winsor, 2015). Darwin was delighted with the distinction, because he saw that common descent could explain both kinds of similarity. In his 1844 sketch he recorded with satisfaction the practice of naturalists.

… how grossly wrong would be the classification, which put close to each other a Marsupial and Placental animal, and two birds with widely different skeletons [swift and swallow]. Relations, such as in the two latter cases, or as that between the whale and fishes, are denominated "analogical," or are sometimes described as "relations of adaption." They are infinitely numerous and often very singular; but are of no use in the classification of the higher groups. (1909, pp.199–200)

…

We shall immediately see on the theory of descent how it comes that there should be "real" and "analogical" affinities; and why the former alone should be of value in classification— difficulties which it would be I believe impossible to explain on the ordinary theory of separate creations. (1909, p. 204)

In the *Origin* Darwin would repeat this point when he said that evolution enables us to understand

the very important distinction between real affinities and analogical or adaptive resemblances. Lamarck first called attention to this distinction, and he has been ably followed by Macleay and others. (1859, p. 427)

Lamarck had proposed two distinct evolutionary processes, the main one being a drive towards increased complexity, the secondary one adaptive. Macleay, a pious creationist, would have been unhappy to find his name next to Lamarck's and he would have rejected Darwin's term “analogical or adaptive,” because Macleay had never used adaptedness as a hallmark of analogy. Naturalists believed that every organism had been created perfectly, with all its parts nicely fitted to their function, so all living things are adapted. What made a relationship analogical in Macleay's system was that the organisms being compared, whether their similar features were obviously useful or not, belonged to different groups. The sperm whale and the blue whale are both adapted to swimming by their streamlined shape, and that shape is part of the many features that show they belong in the family of whales (Cetacea), related by affinity. This entire group has its primary relationship, affinity, to other warm-blooded animals that suckle their young, so Cetacea forms an order in the class Mammalia, but whales also have a secondary relationship, analogy, to the fish class (Pisces), because they share an adaptive feature, their shape. Strickland argued that analogies like this were of no interest to naturalists seeking the natural order because the Creator was forced to follow His own laws of physics when making animals streamlined. For Darwin, the key was not adaptation, since he agreed with other naturalists that most characters were adaptive; the key was recency of common ancestry.

The reason Waterhouse was discussing principles of classification with Darwin in 1843 was that he was working on a paper assessing relationships among the orders of mammals. Waterhouse managed the museum of the Zoological Society of London but hoped to be hired by the British Museum. Although driven by genuine curiosity, he may also have thought that tackling higher groups might enhance his reputation. When Macleay's system was first debated, Waterhouse had tested it by seeking analogies in the insects he was describing (Waterhouse, 1839), but by 1843 he no longer believed in Macleay's system. (The circles printed at the start of Waterhouse's (1843) paper upset Darwin and have misled historians, but they were not lines of affinity, they were symbols of group membership.) Waterhouse agreed with Strickland that groups in the natural system should be based only on affinities.^21^ Many naturalists had proposed connecting links between higher groups, but Waterhouse declared that these usually turned out to be mere analogies, to be ignored in classification: “in proportion as knowledge of the groups and species increases so does the number of supposed links decrease; that is to say, it becomes less and less doubtful as to the group in which an animal should be placed” (Waterhouse, 1843, p. 403). Some favourite examples, such as the similarity of flying squirrels to flying lemurs or of dugongs to dolphins, are obviously adaptive. There was one peculiar case not so easily dismissed, however. Within recent memory, zoologists had classified Australian animals with those they most resemble: the Tasmanian wolf with wolves and the wombat with rodents, but when anatomists studied various marsupial species more thoroughly, other features reinforced the evidence from their reproductive organs, showing that they should form a group distinct from placental mammals. When Owen discovered that a South American rodent called viscacha has a detail in its vagina that slightly resembles marsupials, Waterhouse accepted his judgement that this feature is an affinity rather than an analogy.^22^ Much to Darwin's relief, Waterhouse declared that the affinity of the viscacha is to the entire marsupial group rather than to any particular sub-group within marsupials. The case earned a whole paragraph in the *Origin* (p. 430).^23^

In an 1845 lecture in Oxford, published in the *Philosophical Magazine* in May of 1846, Strickland looked back kindly upon his defeated foe and credited Macleay with being

the first to give us clear definitions on the distinction between AFFINITY and ANALOGY. He applied his views indeed in support of a theory, the *quinary system*, which few naturalists are now disposed to support … but his elucidation of affinities and analogies is not the less valuable on that account … the principles themselves are at the foundation of all sound classification whether in zoology or botany…. (1846a, p. 356)

Strickland went on to propose a change in the meaning of the word *affinity*.

It will thus be seen that every instance of asserted affinity between two organic beings is merely a corollary deduced from an observed affinity between the corresponding organs in each; and though it is not usual to apply the term *affinity* to the similarities between parts, yet as the similarity between the wholes results from the similarities of their parts, the word *affinity* may be as correctly applied to the one as to the other. (1846a, pp. 357–358)

The consequence of this seemingly innocent extension of one key word, long in daily use by naturalists, was that Strickland felt himself equipped to offer advice to men outside of his community who were also in the business of comparing organs.

In works of comparative anatomy it is customary to speak of those members which are essentially equivalent in two organic beings as *analogous* organs, but we shall soon see that the word *analogy* has a very different sense; and as the relation between equivalent organs is one of real *affinity*, and forms the sole ground on which we assert the affinity of the whole beings, we may introduce the adjective *affine* or *homologous* in place of *analogous*, when referring to structures which essentially correspond in different organic beings. (1846a, p. 358)

Britain's leading comparative anatomist lost no time in reacting to this incursion into his territory. Owen stated in the next issue of the journal that Strickland seems

not to have been aware that the term ‘homologous' had been used in the sense he recommends, by comparative anatomists both in this country and abroad for some years past ….

I have been in the habit of defining, in my Introductory Lecture, the terms *homology* and *analogy*, as in the Glossary appended to the Lectures on Invertebrata published in 1843 …. I have always felt and stated that I was merely making known the meaning of a term introduced into comparative anatomy long ago, and habitually used in the writings of the philosophical anatomists of Germany and France. (Owen, 1846, pp. 526–527)

Strickland immediately posted a reply, saying he “was quite aware, though I accidentally omitted to say so, that the word *homologous* had been occasionally used” for “structures which essentially correspond,” and then he claimed (accurately) “that the term *homology* has only been introduced into this country in the last four or five years, and by few if any authors” except for Owen himself.^24^ Strickland then rather naughtily pointed out something Owen knew very well, that the distinction between homology and analogy had been “clearly comprehended by the mighty mind of Aristotle.”^25^ While thanking Owen for “having been the first to introduce the convenient and useful word *homology* in to the language of comparative anatomy in this country,” Strickland insisted that it was naturalists who deserved credit for being the first to clarify the distinction.

Of the two synonymous words *affinity* and *homology*, the latter appears preferable, as being constructed on a similar plan to its antithesis *analogy*. It is to be regretted therefore that the modern zoologists, to whom we are indebted for the first clear definition on this subject did not adopt the term homology instead of affinity, but the latter word is now so well established in systematic zoology, that it is perhaps too late to alter it. (Strickland, 1846b, p. 35)

The words *affinity* and *homology* did not become synonymous at the stroke of Strickland's pen. Most naturalists continued to think of affinity as a relationship between groups, reserving homology for the correspondence of parts of organisms, just as Owen had done. Both words carried on, but their subsequent usage is a story for another day.

## Types, definitions, and archetypes

Continuing his 1843 conversation with Waterhouse in a second letter, Darwin wrote:

[Linnaeus] said the genus gives the characters & not the characters the genus— & yet he leaves undefined what first makes the genus which is to give the characters.— Have you read Whewell's remarks on this subject in his History of the Inductive Sciences? (Burkhardt & Smith, 1990, 2: 378)

William Whewell had quoted with approval Linnaeus's oracular pronouncement, which claimed that each genus is a really existing entity; it is not created by our words, nor by our lists of characteristic features. Linnaeus and Whewell believed that genera were created by God. Darwin liked the aphorism so much that he used it twice in the *Origin*: “Such expressions as that famous one of Linnaeus … the characters do not make the genus, but that the genus gives the characters ….” (1859, p. 413; cf. p. 417).^26^

Whewell was professor of mineralogy at Cambridge University when Darwin was an undergraduate there; Darwin knew and admired this remarkable scientist and polymath. Whewell published his three-volume *History of the Inductive Sciences* in 1837, which Darwin read carefully (di Gregorio, 1990, pp. 865–868; Quinn, 2016, 2017). In this impressive work, Whewell gave an accurate and clear account of the fact that Linnaeus considered his higher groups (class and order) to be artificial and his lower groups (genus and species) natural. Whewell explained that Linnaeus had hoped that in the future his artificial groups would be replaced by natural ones, and Whewell celebrated the fact that botanists like Alphonse de Candolle and zoologists like Cuvier were working toward that end. Naturalists recognized natural groups, Whewell said, by using a sort of “latent instinct.” Darwin wrote in the margin of his copy at this point, “When such expressions are used, it is certain there must be some great hiatus in our knowledge” (di Gregorio, 1990, p. 867). Here our hindsight may intrude and make us think that only branching evolution could fill that hiatus, but Whewell was confident that the discovery of the laws of life would point to the intention of the Creator.

Three years after his *History*, Whewell published two volumes about scientific method. There he explained that naturalists identifying natural groups were wise to ignore the example of mathematics and physics, which are deductive sciences that demand definitions. Indeed the common idea that mathematics provides a model of good reasoning was quite false, he declared.

… the study of Natural History appears to be the proper remedy for this erroneous habit of thought. For in every department of Natural History, the object of our study is *kinds* of things, not one of which kinds can be rigorously defined, yet all of them are sufficiently definite. (1847, vol. 2, p. 370)

Naturalists do not work from a list of characters, he said; what they do is compare a newly discovered form to a known one. Whewell called this the Method of Types. It would be an improvement in liberal education, Whewell argued, if students were given actual plants or animals instead of being limited to reading books. “Thus the study of Natural History, as a corrective of the belief that definitions are essential to substantial truth, might be of great use.” (1847, vol. 2, p. 372).

John Stuart Mill, today far more famous than Whewell, published in 1843 *A System of Logic, Ratiocinative and Inductive; Being a Connected View of the Principles of Evidence and the Methods of Scientific Investigation* (Magnus, 2015; McOuat, 2009). Mill quoted at length Whewell's assertion that zoologists and botanists were right not to define kinds of animals and plants, and then proceeded to contradict him. It is certainly difficult, Mill admitted, to identify all the characters by which we recognize a living kind, and the method of comparing things to a type is often useful; nevertheless, he insisted, a scientific naturalist ought to discover and enumerate characters with which to define a natural group.

In London in the 1850s, Mill was a member of Huxley's circle of friends, while Whewell, who had become Master of Trinity College, Cambridge, was known to be an ally of Owen. Huxley, in his 1854 lecture for the Society of Arts, declared himself indebted to Mill for his ideas about scientific method. Based on the Whewell paragraphs quoted by Mill, Huxley proclaimed:

It is said, in short, that a natural-history class is not capable of being defined — that the class Rosaceæ [the rose family], for instance, or the class of Fishes, is not accurately and absolutely definable, inasmuch as its members will present exceptions to every possible definition; and that the members of the class are united together only by the circumstance that they are all more like some imaginary average rose or average fish, than they resemble anything else.

But here, as before, I think the distinction has arisen entirely from confusing a transitory imperfection with an essential character. So long as our information concerning them is imperfect, we class all objects together according to resemblances which we *feel,* but cannot *define;* we group them round *types,* in short. Thus if you ask an ordinary person what kinds of animals there are, he will probably say, beasts, birds, reptiles, fishes, insects, &c. Ask him to define a beast from a reptile, and he cannot do it; but he says, things like a cow or a horse are beasts, and things like a frog or a lizard are reptiles. You see *he does* class by type, and not by definition. But how does this classification differ from that of a scientific Zoologist? How does the meaning of the scientific class name of "Mammalia" differ from the unscientific of "Beasts"?

Why, exactly because the former depends on a definition, the latter on a type. The class Mammalia is scientifically defined as "all animals which have a vertebrated skeleton and suckle their young." Here is no reference to type, but a definition rigorous enough for a geometrician. And such is the character which every scientific naturalist recognises as that to which his classes must aspire — knowing, as he does, that classification by type is simply an acknowledgment of ignorance and a temporary device.

So much in the way of negative argument as against the reputed differences between Biological and other methods. No such differences, I believe, really exist. The subject-matter of Biological science is different from that of other sciences, but the methods of all are identical …. (1854, pp. 16–18)

Note that the *essential character* Huxley was talking about here did not belong to an organism but to a human activity. He contrasts it with *transitory imperfection*, which is the blemish on biological science that happens whenever a naturalist names a group he cannot yet define. The essential nature of biology is to be a science, Huxley claimed, that is no less exact than mathematics or physics. A consequence of that is that rigorous definition is an element of scientific method. Every “scientific naturalist” understands this, Huxley insists. That term did not describe any taxonomist of Huxley's acquaintance. It prescribed how he wanted natural history to change.

In this public lecture, Huxley was insisting that biological groups were governed by definition rather than by types, yet in his technical publications in the same decade he enthusiastically supported the concept of morphological archetypes. The apparent contradiction is removed when we remember that the types endorsed by Whewell and scorned by Huxley belonged to the practice of taxonomists, who formed an intellectual community with limited overlap with morphologists. From the time of Linnaeus into the early twentieth century, taxonomists evolved the practice of choosing one specimen as the representative of its species (which was called the type but was not necessarily typical), and choosing one species as the type of its genus, to which other species in the genus could be compared (Witteveen, 2016). All these sorts of types were concrete points of reference, either one specimen or one named group, whereas archetypes were abstractions.^27^

Huxley's (1853a) work on the morphology of molluscs, the topic about which Darwin declared himself “so utterly ignorant,” involved comparing his preserved specimens with published descriptions (most of them in German) of a few other gastropods (the group that includes snails, slugs, and sea butterflies) and some cephalopods (the group that includes squid and octopus). His goal of finding the archetype of the Mollusca (one of Cuvier's four animal *embranchements*) was closely modelled on Owen's work on the archetype of the Vertebrata. Both Owen and Huxley operated from the conviction that there were laws of nature that would explain organic form; such laws were secondary causes and could be discovered by careful anatomical observation. These men differed in that Owen was willing to accommodate the religious sensibilities of his audience by associating the archetype with the mind of God, whereas Huxley saw any connection to metaphysics, much less religion, as endangering science's claim to independent authority. He announced this difference boldly.

I think it is now possible to form a notion of the archetype of the Cephalous Mollusca, and I beg it to be understood that in using this term, I make no reference to any real or imaginary “ideas” upon which animal forms are modelled. All that I mean is the conception of a form embodying the most general propositions that can be affirmed respecting the Cephalous Mollusca, standing in the same relation to them as the diagram to a geometrical theorem, and like it, at once imaginary and true. (1853a, p. 50)

**Fig. 1:**
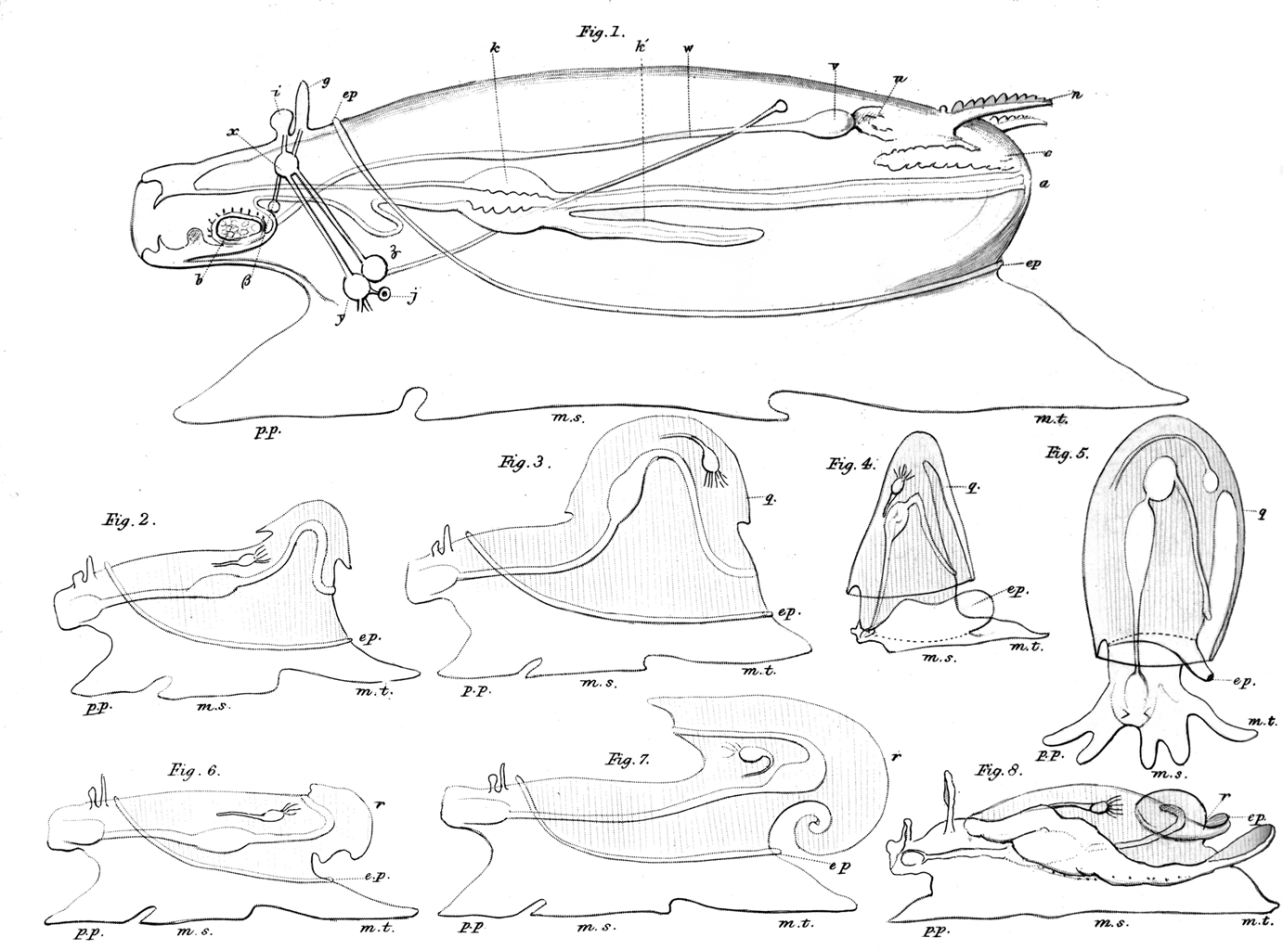
Archetypes of Mollusca conceived and drawn by Thomas Henry Huxley. The top drawing represents the whole group. In the second and third rows, the two drawings on the left show imagined stages of “modification,” and the drawing on the right are archetypes of three molluscan sub-groups: on the second row, Pteropoda and Cephalopoda, and on the third row Heteropoda (Huxley, 1853a, p. 65 and Plate V). This high-definition image, copyrighted by the Royal Society, is used with permission

**Fig. 2:**
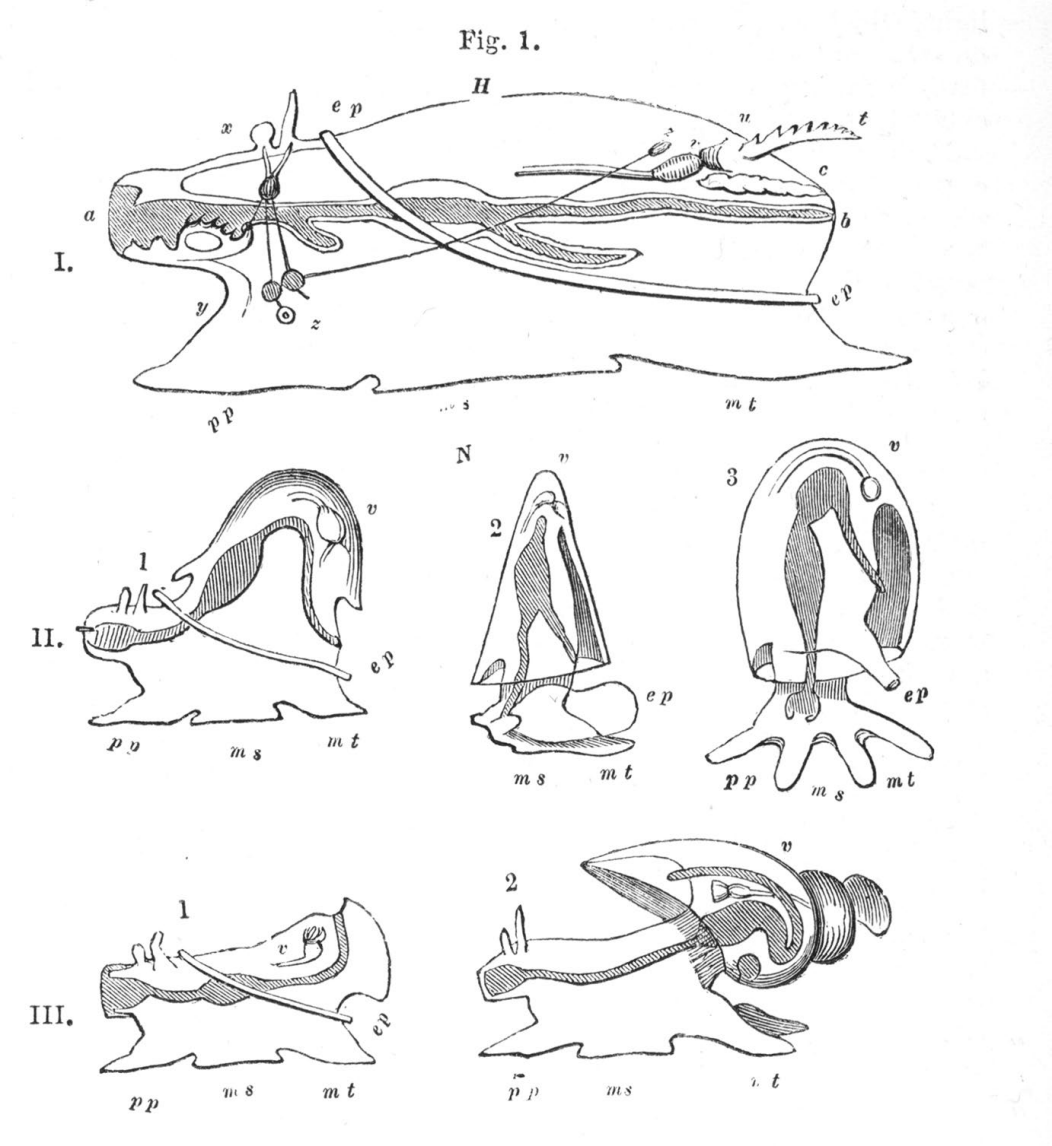
Huxley's archetypal molluscs redrawn for an encyclopedia. Huxley changed his 1853 plate by making the intestines and mantle cavities darker, and he moved the pteropod's heart a bit. On the second row and the third row, he called the first image “hypothetical” (Huxley, 1855, p. 855)

To say that an imaginary conception is nothing like an imaginary idea makes scant sense if lifted out of context, but Huxley was assuming that his readers were familiar with the concept that a mathematical object could be both imaginary and true at the same time (Richards, 1988). He was also sure that his readers understood that his quotation marks around *ideas* referred to Owen's association of archetypes with Plato's *eidos*, the ideal form of which real things are imperfect copies. For Huxley as well as Owen, the archetype was a generalized scheme or common plan, quite literally. It was not just something which could be imagined, it was a physical picture; both men drew their archetypes on paper. Huxley concluded his 1853 study by creating a set of line drawings; the largest was his proposed archetype of Mollusca, then he showed some imagined distortions, and finally some archetypes of molluscan sub-groups (Fig. 1). He simplified those diagrams two years later for an encyclopedia article, commissioned by the publisher Charles Knight (Fig. 2).^28^ Biologists and educators later copied and modified Huxley's (1853a) drawing of the archetype, often presenting it uncritically as if it pictured the ancestor of all molluscs (Lindberg & Ghiselin, 2003).

Huxley insisted in his 1853 study that the main divisions within Mollusca were each referrable to the archetype but not connected to one another. (He knew that Owen was fond of finding transitional forms between types, which might be sufficient explanation for Huxley to venture so far beyond what was then known of molluscan anatomy.)

If … all Cephalopoda, Gasteropoda, and Lamellibranchiata [clams], be only modifications by excess or defect of the parts of a definite archetype, then, I think, it follows as necessary consequence that no anamorphism takes place in this group. There is no progression from a lower to a higher type, but merely a more or less complete evolution of one type. (1853a, p. 63)

At this time, the word *evolution* in biology usually referred to embryological development rather than to historical theories of transformation, but we may still wonder exactly what Huxley meant. Knowing that *Vestiges* had proposed transformation, Huxley made sure to distance himself from that kind of evolution. He was not talking about the history of life, but about the morphological project of comparing abstract forms. This he made clear in a footnote:

It is one thing to believe that certain natural groups have one definite archetype or primitive form upon which they are all modelled; another, to imagine that there exist any transitional forms between them.

Every one knows that Birds and Fishes are modifications of the one vertebrate archetype; no one believes that there are any transitional forms between Birds and Fishes. (1853a, pp. 62n–63n)

His text continued, repeating his own word, *anamorphosis,* for morphological transformation:

It may indeed be a matter of very grave consideration whether true anamorphosis ever occurs in the whole animal kingdom. If it do, then the doctrine that every natural group is organized after a definite archetype, a doctrine which seems to me as important for zoology as the theory of definite proportions for chemistry, must be given up. (1853a, p. 63)

Darwin must have winced when he read this. This was early in their acquaintance, two years before they first discussed Cirripedia. On April 23, 1853, Darwin wrote to Huxley thanking him for this long and technical paper.

The discovery of the type or “idea” (in *your* sense, for I detest the word as used by Owen, Agassiz & Co) of each great class, I cannot doubt is one of the very highest ends of Natural History: & certainly most interesting to the worker out. Several of your remarks have interested me; I am, however, surprised at what you say versus “anamorphism”: I should have thought that the archetype in imagination was always in some degree embryonic, & therefore capable & *generally undergoing* further development. (Burkhardt & Smith, 1990, 5: 133)

In fact for Huxley there was nothing embryonic about the archetype, which was abstracted from adult forms. Darwin followed this with a gentle hint that Huxley should notice a piece of contrary evidence in his own report: “Is it not an extraordinary fact, the great difference in the position of the heart in different species of Cleodora?” (Burkhardt & Smith, 1990, 5: 133) Here was a structural feature that varied from one species to another within the same genus.^29^ This ought to have been enough to destroy Huxley's claim about types within Mollusca, because the position of the heart in relation to the gills was supposed to be a character indicative of a higher division of gastropods.^30^Huxley made his understanding of archetype even clearer in the encyclopedia article.

By the Common Plan or Archetype of a group of animals we understand nothing more than a diagram, embodying all the organs and parts which are found in the group, in such a relative position as they would have, if none had attained an excessive development. It is, in fact, simply a contrivance for rendering more distinctly comprehensible the most general propositions which can be enunciated with regard to the group, and has the same relation to such propositions as the diagrams of a work on mechanics have to actual machinery, or those of a geometrical work to actual lines and figures. We are particularly desirous to indicate the sense in which such phrases as Archetype and Common Plan are here used; as a very injurious realism— a sort of notion that an Archetype is itself an entity— appears to have made its way into more than one valuable anatomical work. It is for this reason that if the term Archetype had not so high authority for its use, we should prefer the phrase 'Common Plan' as less likely to mislead. ([Huxley], 1855, p. 856)

The simple four-letter word *plan*, also used by surveyors and architects for an important piece of paper, seemed to Huxley to be a way to avoid metaphysics. Before long he stopped using the word *archetype*, putting all his money on the word *plan*. Consider that 1857 document which Darwin called “simply the very best Resume by far, on the whole Science of Natural History, which I have ever seen ….” (Burkhardt & Smith, 1990, 6: 505) This document, we must remember, was not an original technical report like the Mollusca article, but was meant as an introduction aimed at students new to biology. Huxley wrote that after an anatomist had identified the structures common to antelopes, sheep, oxen, deer, giraffes, and camels, he could list their common characters, and then find it easy to “make a drawing” which

would stand in precisely the same relation to the group … as the ground plan of a single house does to the street which the architect means to build of houses of that size and general form. The superstructure of each house may, if the architect pleases, be totally different in style, without in any way interfering with his general plan; and similarly, in each particular ruminant, the common plan is preserved, while the details of the “elevation,” the size, the figure, the proportions, the ornamentation in the way of color and horns, vary to an immense extent ….

It is most important, however, not to form a wrong idea as to the real import of these “common plans.” We must regard them simply as devices by which we render more clear and intelligible to our own minds the great truth that the parts of living bodies are associated together according to certain definite laws.

…

But it is obvious that if animals and plants were not constructed upon common plans, it would be impossible to throw them into groups expressive of their greater or less degree of resemblance, such as those of the natural classification. In fact, the doctrine of “common plan” and of “natural classification” are but two ways of expressing the great truth, that the more closely we examine into the inner nature of living beings, the more clearly do we discern that there is a sort of family resemblance among them all, closer between some, more distant between others, but still pervading the whole series. (Huxley, 1869, pp. 366–369)

Even though Darwin told Huxley that “Your metaphors & explanations strike me as *admirable*” (Burkhardt & Smith, 1990, 6: 505), Darwin's own practice was to be leery of metaphors. His original discovery had emerged from identifying “relationship” as metaphorical unless it applied to literal kinship, meaning the parent-to-offspring connection. He was careful to spell out the kinds of physical events in nature that his novel metaphorical sense of *selection* stood for. In his 1857 reply to Huxley's census letter, Darwin spoke of “the plan on which the Creator has worked” even though he knew that Huxley abjured that meaning of *plan*. Two years later when Darwin wrote in the *Origin*, “It is so easy to hide our ignorance under such expressions as the 'plan of creation,' 'unity of design,' &c., and to think that we give an explanation when we only restate a fact,” (p. 482) he may have intended a subtle reprimand to Huxley.

## The difficulty remains the same

The fundamental structure of Darwin's book did not change from the time he wrote out sketches of his argument in 1842 and 1844, nor did his view of classification.^31^ What he had believed ever since his 1837 notebook drawing of an evolutionary tree is stated in Chapter XVIII of the *Origin:*

…that the natural system is founded on descent with modification; that the characters which naturalists consider as showing true affinity between any two or more species, are those which have been inherited from a common parent, and in so far, all true classification is genealogical; that community of descent is the hidden bond which naturalists have been unconsciously seeking, and not some unkown plan of creation, or the enunciation of general propositions, and the mere putting together and separating objects more or less alike. (1859, p. 420)

He predicted that after evolution is accepted, “Systematists will be able to pursue their labours as at present” (p. 484), because he did not claim that believing “all true classification is genealogical” gave taxonomists any new operating principles or rules to work by. When discussing the principles of classification with Waterhouse in 1843, Darwin had admitted that under his theory, “the difficulty of ascertaining true relationship, i.e. a natural classification, remains just the same …” (Burkhardt & Smith, 1990, 2: 376).^32^ In the course of their search for the natural system, taxonomists had learned some hard lessons. What did not work, they found, was expecting a regular pattern, requiring perfect definitions, and choosing characters by their importance. Darwin was satisfied that his theory could explain why groups are irregular and why no characters are special, and those explanations he counted as evidence supporting his theory. Yet when he envisioned how biology would look when evolution is accepted, Darwin did not write “Our classifications will come to be genealogical.” He well knew the difficulties with which taxonomists already struggled, and so he wrote, “Our classifications will come to be, as far as they can be so made, genealogical.” (1859, p. 486).

The story of Huxley's public defence of evolution is one of the best known in the history of science. Instead of claiming that Darwin was right, he made the issue about protecting science from religious control. With respect to evolution, Huxley's ideas differed from Darwin's in a number of significant ways: he was lukewarm about natural selection and he held that the origin of species lacked experimental proof. Although he stopped using the word archetype, Huxley never abandoned the metaphor of plan. Nor did he ever change his view of what is wanted in proper science: that facts of structure should be recorded independent of theory (Lyons, 1995; Richmond, 2000; Winsor, 1985). Huxley's words probably had at least as much influence on nineteenth century biology as anything Darwin wrote, not only because of the wide readership of Huxley's essays, but because of his influence on education. His introductory textbooks, and those written by his students, presented facts of physiology and anatomy as faits accomplis, and mostly ignored evolution.

Darwin never claimed that classification must be based upon evolution, because he knew that before 1859 taxonomists had made good progress toward discovering the natural system. He simply expected that people who believe in evolution would naturally prefer taxonomic groups to express genealogy whenever genealogy can be inferred. By 1868 Huxley came to agree with this (DiGregorio 1984, p. 78). Other biologists have likewise fulfilled Darwin's expectation, but there have been significant exceptions, especially among botanists (Mishler, 2014; Sterner, 2014; Winsor, 1995). Beginning in the 1970s some followers of Willi Hennig warn that reading a cladogram (a diagram of homologous characters) as a phylogenetic tree may be reasoning in a circle, since homology has been redefined as similarity due to evolution (Brady, 1985). Although most biologists treat taxonomic groups and evolutionary history as unproblematic synonyms, there are some who insist that patterns of similarity should be kept distinct from the process that explains them (Brower, 2019).

In an early draft of this paper, I called the disagreement a *quarrel*. I am grateful to the anonymous reviewer who objected that this word is too strong. If *quarrel* calls to mind two scrappy dogs, it would be quite wrong, for these men understood the rules of civilised behaviour. What Darwin wrote to Huxley was merely “I fear we shall split.” It was to his close friend Hooker that Darwin confessed the strength of his feelings. If *quarrel* suggests a lovers' spat, over a trivial matter soon forgotten, that also would make it the wrong word. The connection between biological classification and evolution was then, and remains, an important issue, and their language shows that Huxley and Darwin both thought it was important. My reviewer also suggested that Darwin's “I would sooner die than give up” was only meant tongue-in-cheek. The phrase is of course hyperbole, but I have no doubt that Darwin meant that he would never alter his view on this point. He was saying that since Huxley's feelings on the question were just as strong as his own, rational debate would be futile. While we may congratulate Darwin on his emotional intelligence, it left the issue obscure and unexplored.

## Data Availability

Not applicable.

## Footnotes

1 Most people who quote Theodosius Dobzhansky's polemic understand it as a challenge to creationism, which is indeed how he used it in 1973, but when he invented this rhetorical claim in 1964, he aimed it at a different foe. He was complaining that molecular biologists were disrespecting organismic biologists.

2 Many writers dramatise the narrative by saying he kept his theory secret and delayed publishing, but these claims are exaggerated (van Wyhe, 2007).

3 A great many outstanding scholars have analysed the lives and work of these men (Ospovat, 1981; Burkhardt & Smith, 1990; Desmond, 1982, 1994, 1997; Rupke, 1994; Browne, 1995, 2002). Although I give no citations to historical facts that are widely available, I am deeply aware that history does not write itself. Many elements in this story have been told already (di Gregorio, 1982a, b; Lyons, 1995). All of Darwin's publications and most of Huxley's are available online (Blinderman & Joyce; van Wyhe 2002-; Biodiversity Heritage Project).

4 To read private letters which were composed long ago is the closest we can come to travelling back in time. Unlike documents written for publication, which make up the official record of science, the transcription of such manuscripts allows us to access thoughts that may have been important to the writer but which they chose not to make public. Nor is writing limited to two categories, private and public. Outside of their published writings, people have thoughts they share with no one (but might enter in a diary); other thoughts they express to their closest confidantes; still others they share with colleagues (perhaps in an after-dinner speech). Most such private words are ephemeral, so anyone who cares about history should support archivists, those professional scholars who make such sources of information available.

5 Although the system is now called “hierarchical,” and the term “Linnaean hierarchy” is now common, nineteenth-century naturalists used those terms rarely, if at all. Darwin said that the subordination of group within group is a “truly wonderful fact” (1859, p. 128) “which, from its familiarity, does not always sufficiently strike us” (1859, p. 413).

6 A second meaning of *higher* was still common. The old idea of a great chain of being put humans at the top and worked down through other animals and plants towards whatever seemed simpler. This belief was important to many naturalists, and its significance in the history of taxonomy deserves attention, but it played no role in this particular discussion between Huxley and Darwin.

7 The noun *group* had and still has two meanings; sometimes the writer meant a kind of category in a classification, such as *order* and *genus*, but at other times a particular concrete instance was meant, such as *barnacles* or *Mammalia*. Twentieth-century biologists tried to eliminate the ambiguity by coining the word *taxon* (plural *taxa*) for the concrete sense, but some biologists are using *taxon* to mean a category, so I have chosen to stick with *group* and let the context show what is meant. I am indebted to Michael Ghiselin (1997) for my own understanding of this crucial distinction.

8 Although *human invention* and *man-made* should be synonymous with *artificial*, I have avoided this word because in the nineteenth century, naturalists used *artificial* for a classification based on a few characters, as opposed to one based on many characters, which most naturalists recognized was also created by humans.

9 I have deleted the lone quotation mark that Darwin put before the word *Natural*.

10 Aiming at precision, the system equipped the army of enumerators with pre-set questions. Among other puzzles, officials had to decide whether to count British sailors on shipboard (Higgs, 2005). Huxley, still in the Navy, had just disembarked from the *Rattlesnake* and was staying with his brother on census night; whether the captain remembered to count him, I do not know.

11 I suspect that a direct line of descent could be traced back to the Abbé de Condillac's 1749 *Traité des Systêmes.*

12 Darwin often wrote “w^d^” and “s^d^,” which I have changed to “would and “should.”

13 This was his “Explanatory Preface,” “Explanatory Introduction,” or “Principles and methods” (1857b, 1865, 1869, 1901). He was doubtless well along in writing it in late September or early October when he sent Darwin his census letter. Huxley's published version stated “As Cuvier has well expressed it, modern classification endeavors to throw the facts of the structure of living beings into the fewest possible general propositions.” (1869, p. 369)

14 I have replaced Darwin's “strikee” with “strike.”

15 Darwin told Hooker (Burkhardt 6: 516) that he had read Huxley's text in proof (the first step of the printing process).

16 When he inserted an extra chapter in the 6th edition of 1872, this became Chapter XIV.

17 This is close to the heart of the issue: how closely does resemblance actually track genetic relationship? What made the first part so ridiculous, when he proposed “a long course of modification”? The problem hinges on the circularity of naturalists' definition of affinity, which was never a synonym for resemblance (Winsor, 2015).

18 Labels for disciplines, practices, styles of thought, and communities of practice are notoriously hard to pin down, because their meanings shift in time and place. I have been inspired by Lynn Nyhart's chapter about Germany zoology, “Rearranging the sciences of animal life, 1845-1870” (1995, pp. 65-102).

19 The term *ex professo* did not mean “as a professor” or even “as a professional” but “as an expert.” Huxley was referring to the fact that prior to the Cirripedia volumes, all Darwin's writings were on natural history.

20 In Owen's lectures on invertebrates, he says of arthropods: “The jaws, when present, are lateral, and move from without inwards, and not from above downwards.” (1843, p. 14) The qualification “when present” signalled his awareness of jawless forms.

21 At the same meeting of the British Assoiation for the Advancement of Science at which Waterhouse presented his ten orders of mammals, Strickland unfurled his large chart of affinities of birds, and reported that he and Waterhouse thought much alike (Winsor 2015, 3–4:179–181). Henri Milne-Edwards was also working on patterns of affinity in Paris; he said that Waterhouse's ideas were similar to his and that Macleay had done a great service to zoology by calling attention to *affinitės collatėrales*, which, unlike analogies, were real affinities (Milne-Edwards, 1844, p. 65 and p. 78).

22 The viscacha (*Lagostomus maximus*) has its vagina partly divided by a septum; most marsupials have two vaginas. Darwin, having seen these animals in Argentina, called them “bizcacha.”

23 Darwin's discussion of the case was ignored by everyone, perhaps because few Europeans had seen a viscacha. Many biologists throughout the nineteenth and twentieth century viewed any links between groups as supportive of evolution, but Darwin recognized that for well-defined higher groups, common ancestors belong in the distant past. I am grateful to Gunther Wagner for confirming that Owen and Waterhouse were mistaken to treat the divided vagina of Lagostomus as homologous to the paired vagina of marsupials.

24 Although Owen defined “homologue” (coupled with “analogue”) in 1843, use of the word “homology” by British biologists dates from 1848, after the appearance of his book *On the Archetype and Homologies of the Vertebrate Skeleton* (Wood 1995).

25 Aristotle's *Historia Animalium* begins by saying that part of animals in the same class are the same, while parts doing the same thing in different classes are analogous.

26 Linnaeus wrote (in *Philosophia Botanica*, Stockholm, 1751, aphorism number 169): “*Scias Characterem non constituere Genus, sed Genus Characterem. [Scias] Characterem fluere a Genere, non Genus e Charactere. [Scias] Characterem non esse, ut Genus fiat, sed ut Genus noseatur.*” Whewell wrote “the Character does not constitute the Genus, but the Genus the Character;—the Character is necessary, not to make the Genus, but to recognize it.” The English word “character” means, today and to taxonomists in the nineteenth century, a single useful feature, but for that, Linnaeus used the Latin word *nota*. Linnaeus's word *character* meant a number of *notae*, so when Darwin used the English plural “characters," he captured Linnaeus's meaning.

27 This oversimplifies a wider story of the word *type*. Another important sense arose when Huxley developed a way to teach introductory biology called the “type method,” which used a series of individual exemplary creatures (frog, rat, yeast) rather than surveying the whole diversity of life. The pedagogy was “practical” because students were supposed to see and dissect the specimen themselves.

28 It was Mario di Gregorio (1984) who first identified this anonymous entry as Huxley's, as it certainly is.

29 The genus Cleodora is now Creseis.

30 Huxley had called attention to a fact he had found in the literature (he had not collected *Cleodora spp.* himself): “In this one genus, then, we have every transition from the prosobranchiate to the opisthobranchiate type of organization.” (1853a, p. 42) The differences were between species, not within a species. The taxonomic value of that character remained high in the nineteenth century but dissolved in the twentieth.

31 Those who study Darwin closely know that he made a significant improvement in his theory in the 1850s, when he expanded his view of variation and imagined how competition could drive divergence (Ospovat, 1981). These important insights changed neither the structure of his argument nor his perception that the natural system expressed genealogy.

32 I have added punctuation to this fragment from Darwin's letter.

## References

[CR1] Anon. (1854). Meetings for the ensuing week. Journal of the Society of Arts.

[CR2] Barlow N (1958). The autobiography of Charles Darwin.

[CR3] Blinderman, C., & Joyce, D. (Eds.) (1998). *The Huxley File.* https://mathcs.clarku.edu/huxley/

[CR4] Brady RH (1985). On the independence of systematics. Cladistics.

[CR5] Browne J (1995). Charles Darwin: Voyaging: A biography.

[CR6] Browne J (2002). Charles Darwin: The power of place: Volume II of a biography.

[CR7] Brower AVZ (2019). Background knowledge: The assumptions of pattern cladistics. Cladistics.

[CR8] Burkhardt, F. & Smith, S. (Eds.) (1990). *The correspondence of Charles Darwin.* Cambridge University Press. https://www.darwinproject.ac.uk

[CR9] Cuvier, G. (1800). *Leçons d'anatomie comparée.* Paris.

[CR10] Cuvier, G. (1854). *The animal kingdom, arranged after its organization, forming a natural history of animals, and an introduction to comparative anatomy*. E. Blyth, R. Mudie, G. Johnston, J. O. Westwood, & W. B. Carpenter (Eds.). W. S. Orr.

[CR11] Dana, J. D. (1853). *On the classification and geographical distribution of Crustacea: From the report on Crustacea of the United States Exploring Expedition, under Captain Charles Wilkes, U.S.N. during the years 1838–1842.* Philadelphia.

[CR12] Darwin, C. R. (1839). *Narrative of the surveying voyages of His Majesty's Ships* Adventure *and* Beagle *between the years 1826 and 1836, describing their examination of the southern shores of South America, and the Beagle's circumnavigation of the globe. Journal and remarks. 1832–1836.* Henry Colburn.

[CR13] Darwin CR (1851). A monograph on the fossil Lepadidae, or, pedunculated cirripedes of Great Britain.

[CR14] Darwin CR (1851). A monograph on the sub-class Cirripedia, with figures of all the species: The Lepadidae, or, pedunculated cirripedes.

[CR15] Darwin CR (1854). A monograph on the sub-class Cirripedia with figures of all the species: The Balanidae, (or sessile cirripedes); the Verrucidae.

[CR16] Darwin CR (1859). On the origin of species by means of natural selection, or the preservation of favoured races in the struggle for life.

[CR17] Darwin F (1909). The foundations of the Origin of Species: Two essays written in 1842 and 1844.

[CR18] Desmond A (1982). Archetypes and ancestors: Palaeontology in Victorian London 1850–1875.

[CR19] Desmond A (1989). The politics of evolution: Morphology, medicine, and reform in radical London.

[CR20] Desmond A (1994). Huxley: The devil's disciple.

[CR21] Desmond A (1997). Huxley: Evolution's high priest.

[CR22] Desmond A, Moore J (1991). Darwin: The life of a tormented evolutionist.

[CR23] Endersby J, Curry HA, Jardine N, Secord JA, Spary EC (2018). Descriptive and presecriptive taxonomies. Worlds of natural history.

[CR24] di Gregorio MA (1982). In search of the natural system: Problems of zoological classification in Victorian Britain. History and Philosophy of the Life Sciences.

[CR25] di Gregorio MA (1982). The dinosaur connection: A reinterpretation of T. H. Huxley's evolutionary view. Journal of the History of Biology.

[CR26] di Gregorio MA (1984). T. H. Huxley's place in natural science.

[CR27] di Gregorio MA (1990). Charles Darwin's marginalia.

[CR28] Ghiselin MT (1997). Metaphysics and the origin of species.

[CR29] Higgs E (2005). Making sense of the census revisited: Census records for England and Wales 1891–1901.

[CR30] Hitchcock, D. (2017). Is there an argumentum ad hominem fallacy? In *On reasoning and argument: Essays in informal logic and on critical thinking*, pp. 409–419. https://link.springer.com/chapter/10.1007%2F978-3-319-53562-3_26

[CR31] Huxley L (1900). *Life and letters of Thomas Henry Huxley*, 2 vols.

[CR32] Huxley TH (1849). On the anatomy and affinities of the family of the medusae. Philosophical Transactions of the Royal Society of London.

[CR33] Huxley TH (1853). On the morphology of the Cephalous Mollusca, as illustrated by the anatomy of certain Heteropoda and Pteropoda collected during the voyage of H.M.S. Rattlesnake in 1846–50. Philosophical Transactions of the Royal Society of London.

[CR34] Huxley TH, Henfrey A, Huxley TH (1853). Fragments relating to philosophical zoology, selected from the work of K. E. von Baer. Scientific memoirs, selected from the transactions of foreign academies of science, and from foreign journals: Natural history.

[CR35] Huxley, T H. (1854). *On the educational value of the natural history sciences*. London: Van Voorst. Available online from the British Library and Google. It was reprinted in his 1870 *Lay Sermons,* and in vol. 3 of his *Collected Essays: Science and Education*, and again in Cyril Bibby, *T. H. Huxley on Education: A selection from his writings*.

[CR36] Huxley TH, Knight C (1855). Mollusca. The English cyclopædia: Natural history.

[CR37] Huxley TH (1857). Lectures on general natural history. Medical Times and Gazette.

[CR38] Huxley, T. H. (1857b). [*Explanatory preface to the fossil collection in the Museum of Practical Geology*]. Leonard Huxley stated (1900, Vol. 1, p. 159) that this was published in 1857, but no copy has yet been found. It was published in 1865, 1869, and 1901.]

[CR39] Huxley TH, Etheridge R (1865). Explanatory introduction. A catalogue of the collection of fossils in the Museum of Practical Geology.

[CR40] Huxley, T. H. (1869). *Principles and methods of palæontology*. Annual Report of the Board of Regents of the Smithsonian Institution, pp. 363–388.

[CR41] Huxley TH, Foster M, Lankester ER (1901). Explanatory preface to the catalogue of the palæontological collection in the Museum of Practical Geology. The scientific memoirs of Thomas Henry Huxley.

[CR42] Layton D (1973). Science for the people: The origins of the school science curriculum in England.

[CR43] Lindberg DL, Ghiselin MT (2003). Fact, theory and tradition in the study of molluscan origins. Proceedings of the California Academy of Sciences.

[CR44] Lyons SL (1995). The origins of T. H. Huxley's saltationism: History in Darwin's shadow. Journal of the History of Biology.

[CR45] Magnus PD (2015). John Stuart Mill on taxonomy and natural kinds. HOPOS: The Journal of the International Society for the History of Philosophy of Science.

[CR46] McOuat GR (1996). Species, rules and meaning: The politics of language and the ends of definitions in 19^th^ century natural history. Studies in History and Philosophy of Science.

[CR47] McOuat GR (2009). The origins of natural kinds: Keeping “essentialism” at bay in the Age of Reform. Intellectual History Review.

[CR48] Milne-Edwards H (1844). Considėrations sur quelques principes relatif à la classification naturelle des animaux. Annales des sciences naturelles, Series.

[CR49] Mishler B, Hamilton A (2014). History and theory in the development of phylogenetics in botany. The evolution of phylogenetic systematics.

[CR50] Nyhart LK (1995). Biology takes form: Animal morphology and the German universities, 1800–1900.

[CR51] Ospovat D (1981). The development of Darwin's theory: Natural history, natural theology, and natural selection, 1838–1859.

[CR52] Owen, R. (1843). *Lectures on the comparative anatomy and physiology of the invertebrate animals.* London. PMC5199594 30164901

[CR53] Owen R (1846). Observations on Mr Strickland's article on the structural relations of organized beings. London, Edinburgh and Dublin Philosophical Magazine and Journal of Science, Series 3.

[CR54] Owen, R. (1849). On the nature of limbs. In R. Amundson (Ed.), *On the nature of limbs: A discourse.* University of Chicago Press, 2007.

[CR55] Owen, R. (1855). *Lectures on the comparative anatomy and physiology of the invertebrate animals* (2 ed.). London.

[CR56] Quinn A (2016). William Whewell’s philosophy of architecture and the historicization of biology. Studies in History and Philosophy of Science Part C: Studies in History and Philosophy of Biological and Biomedical Sciences.

[CR57] Quinn A (2017). Whewell on classification and consilience. Studies in History and Philosophy of Science Part C: Studies in History and Philosophy of Biological and Biomedical Sciences.

[CR58] Richards JL (1988). Mathematical visions: The pursuit of geometry in Victorian England.

[CR59] Richmond ML (2000). T. H. Huxley's criticism of German cell theory: an epigenetic and physiological interpretation of cell structure. Journal of the History of Biology.

[CR60] Rieppel O, Hamilton A (2013). The early cladogenesis of cladistics. The evolution of phylogenetic systematics.

[CR61] Rupke NA (1994). Richard Owen: Victorian naturalist.

[CR62] Sterner B, Hamilton A (2014). Well-structured biology. The evolution of phylogenetic systematics.

[CR63] Stevens PM (1994). The development of biological systematics: Antoine-Laurent de Jussieu, nature, and the natural system.

[CR64] Strickland HE (1840). Observations upon the affinities and analogies of organized beings. Magazine of Natural History.

[CR65] Strickland HE (1841). On the true method of discovering the natural system in zoology and botany. Annals and Magazine of Natural History.

[CR66] Strickland HE (1846). On the structural relations of organized beings. London, Edinburgh and Dublin Philosophical Magazine and Journal of Science, Series 3.

[CR67] Strickland HE (1846). On the use of the word homology in comparative anatomy. London, Edinburgh and Dublin Philosophical Magazine and Journal of Science, Series 3.

[CR68] van Wyhe, J. (Ed.) (2002). *The Complete Work of Charles Darwin Online.* http://darwin-online.org.uk/

[CR69] van Wyhe J (2007). Mind the gap: Did Darwin avoid publishing his theory for many years?. Notes and Records of the Royal Society.

[CR70] van Wyhe J (2019). Why there was no “Darwin's bulldog”: Thomas Henry Huxley's famous nickname. The Linnean.

[CR71] Waterhouse GR (1839). Descriptions of some new species of exotic insects. Transactions of the Entomological Society of London.

[CR72] Waterhouse GR (1843). Observations on the classification of the Mammalia. Annals and Magazine of Natural History.

[CR73] Whewell W (1847). The philosophy of the inductive sciences, founded upon their history.

[CR74] Winsor MP (1969). Barnacle larvae in the nineteenth century: A case study in taxonomic theory. Journal of the History of Medicine and Allied Sciences.

[CR75] Winsor MP, Weinstock JM (1985). The impact of Darwinism on the Linnaean enterprise, with special reference to the work of T. H. Huxley. Contemporary perspectives on Carl von Linné.

[CR76] Winsor MP (1976). Starfish, jellyfish, and the order of life: Issues in nineteenth-century science.

[CR77] Winsor MP (1991). Reading the shape of nature: Comparative zoology at the Agassiz Museum.

[CR78] Winsor MP (1995). The English debate on taxonomy and phylogeny, 1937–1940. History and Philosophy of the Life Sciences.

[CR79] Winsor MP (2009). Taxonomy was the foundation of Darwin's evolution. Taxon.

[CR80] Winsor, M. P. (2015). Considering affinity. *Endeavour* 39(1), 69–79; 39(2), 116–126; 39(3–4), 179–187. 10.1016/j.endeavour.2014.06.002 25190074

[CR81] Witteveen J (2016). Suppressing synonymy with a homonym: The emergence of the nomenclatural type concept in nineteenth century natural history. Journal of the History of Biology.

[CR82] Wood SW (1995). The first use of the terms “homology” and “analogy” in the writings of Richard Owen. Archives of Natural History.

